# P261: Study of hospital associated infections (HAI) at tertiary hospital in India; economic implication for developing countries

**DOI:** 10.1186/2047-2994-2-S1-P261

**Published:** 2013-06-20

**Authors:** S Satpathy, A Chaudhry, SK Gupta, A Kapil

**Affiliations:** 1 Hospital administration, All India institute of medical sciences, New Delhi, India; 2 Microbiology, All India institute of medical sciences, New Delhi, India

## Introduction

Over 1.4 million people worldwide suffer from HAI at any given time. Hospital wide prevalence of HAI varies from 5.7% to 19.1%, witha pooled prevalence of 10.1%. Surgical site infections are most frequent in developing countries, with incidence rates from 1.2 to 23.6 per 100 surgeries. The adverse impact includes increased mortality and morbidity, and compromised quality of life of the patient and family [1]. This study focuses on the economic consequences and social burden of HAI cases in a tertiary level hospital.

## Objectives

To calculate the HAI incidence rate among surgical post-operative patients.

To compute the total cost incurred by hospital due to HAI in these patients.

To ascertain the out of pocket (OOP) expenditure by patients due to HAI.

## Methods

147 post-operative patients (47- cases with HAI and 100-controls, without HAI) meeting inclusion criteria, matched for sex ratio and type of surgeries were included in the final sample. Method of data collection included observations, patients record, microbiology lab reports, interview with attending nurses, and questionnaire for patients/family attendants. Financial burden on the hospital and patient was calculated following standard costing protocols.

## Results

HAI incidence rate was 6.01% during 3 months study period. Mean age of patients with and without HAI was 48 and 43 years. Both the groups differed significantly in terms of average length of stay (ALS), total cost of hospital stay, cost of medicines and consumables, cost of boarding and lodging of attendant. Other qualitative and quantitative findings are discussed contextually with greater implications for hospitals and society in developing countries.

## Conclusion

The economic implication of increased ALS in the study group by 2-4 times was a significant increase in the total cost incurred to both the hospital and patient, further compounded by the social burden of one attendant having to stay with the patient, resulting in notional loss of income. Considering the low per capita income and high out of pocket expenditures in India, HAIs place an even greater burden on the already scarce resources.

## Competing interests

None declared
